# Letter from H. J. McKellops, Paris

**Published:** 1865

**Authors:** H. T. McKellops

**Affiliations:** Paris


### Correspondence.

PARIS, September, 25th, 1865.

*My Friend Taft:*—I hope you will excuse me, but I can not let this opportunity pass without writing you a few lines in relation to our profession and the country; as this is my first, I hope you will make all due allowances. Upon my arrival in London I paid a visit to the profession in this most wonderful city, and gathered up all the information that I thought would be interesting to you. I have been kindly received by all the profession, and visited with the greatest pleasure the Dental Hospital, in which more than twelve thousand patients have been gratuously taken care of in the course of the present year. The operating room of this Institute has six chairs and the necessary dental furniture to make it complete, and furnished with instruments used in every operation. There is one Demonstrator for each day, one hour and a half, and from ten to twenty students. The patients are received in one large room and there admitted by an attendant whose business it is to conduct them in turns to the operating room, where they are received by the Demonstrator, who explains what is to be the operation and how it is to be done. This Institute is supported by voluntary contributions of the public. Enclosed you will find a report of this useful establishment: in the same building is held the meetings of the Dental Society, and I think, also, the College; I had also the opportunity of seeing and admiring the Dental Museum of the Society; there were many very fine collections. The one that attracted my attention most was that presented by Dr. Tomes; it was very interesting, and must have required many years to be brought to this high degree of perfection. Dr. Tomes is a most estimable and pleasant gentleman, and my visit has been, to me, as agreable as interesting. I also saw Dr. Edwin Serconde, a young man who is gaining a high reputation, Dr. Ballor, Rogers, and a great number of others.

Mr. Ash & Sons, Dental Depot is really very fine, and the profession of London ought to be very proud of it, as it is the largest I have seen on this side of the ocean. It is well supplied with teeth, instruments, dental furniture of all kinds, (except chairs,) anatomical and miscroscopic preparations; his manufactory of teeth is very large, and the teeth are excellent and very strong; the recommendations of the teeth are as follows: They can be ground and polished, and are not porous, and have a great deal of strength, and can stand any amount of fire; I speak of plain teeth, as they are more generally used; gum teeth are very little employed. As to gold foil, I find that none can be compared with J. B. Dunlevy's, of Pittsburgh, Pennsylvania; it may be I am partial to it, but, nevertheless, truth is mighty, and must prevail. London is a very good place for men of our profession, it needs a few "American Dentists" to stir them up and to take the rust off the old fogyism that exists here. Amalgam is chiefly used for filling, and the reason is given that the people will not submit and do not appreciate gold, every one wishing to have the operation done quickly. As an illustration of that, I was told of a lady who had one front tooth decayed on its front surface, and which wanted to be filled, she refused to have it filled with gold for fear of it being seen, and preferred gutta percha, though the operation was to be renewed every two weeks, and each sitting costs her one pound; the fees as you may see are good, (one pound for extracting, one pound for filling, and one pound for consultation,) the largest practice I have heard of in London was Dr. Rogers & Sons, ($90,000) ninety thousand a year.

The city and sight seeing has also had a great share of my attention, and I found great interest in my visits to all the buildings whose names are known and familiar to those who read English History, viz: the Tower of London, Westminster Abbey, Windsor Castle, the House of Lords or Parliament, etc. However, Paris, in that respect, is the first place of the world, and no written description can give an exact idea of the magnificence of the buildings and monuments with which the city is adorned. Imagine several miles of large and beautiful houses all of them built in the same style and of the same materials, and splendidly enough to rival with any palace.

The *Boulevards* which are the fashionable streets of the city are planted with trees which throw an agreeable shade; pretty gardens are scattered here and there in the most populous parts and give to the laboring classes a good opportunity to breathe a delicious atmosphere perfumed with exotic plants and kept cool by artificial fountains, which are very nice and artistic. But fashionable classes and foreigners may enjoy those pleasures, and at a greater extent by riding along the *Champs Elysées* so worthy of their fabulous name, having on the right hand the *Arc de Triamphe* one of the most beautiful monuments of France, they reach that celebrated *Bois de Bologne*, which is, as near as I can describe it, a fairy land, with its beautiful woods, fine drives for miles, with its artificial lakes covered with boats and water fowls, and the many islands which dot it here and there, and their *cascades*, *caves*, *Jardin d'acclimation*, or Zoological Museum, cencerts, and restaurants, all assembled there to attract the sight and delight the senses. In town, historical palaces, paintings, museums, splendid churches, have by turn attracted my eyes and excited my admiration; but the one which filled my soul with the greatest veneration and respect was the tomb of Napoleon I, at the *Hotel des Invalids.* It seems indeed that the whole glory and genius of France comes out of this powerful and glorious grave, lavishing the light of their splendid rays over all Europe. It is a fact, indeed, that this great man, the greatest of many centuries has left in Italy, Germany, Belgium and Spain the mark of each one of his victorious steps and of his powerful genius of legislation and administration.

Versailles also deserves to be spoken of; the splendid palace and its magnificent collection of paintings, as the park and its numerous fountains and statues that adorn its grounds are all grand in the extreme. In this I had the pleasure of paying my respects to Dr. Bruster, the oldest dentist of this country, and I assure you I found him one of the most agreeable men I ever met; he lives at Versailles upon the money he made from a long and worthy practice. Though the sight seeing has almost fascinated my mind, I soon paid visits more important to me. I saw Dr. Yage, who has been practising here for twelve years; Dr. Parmley from New Orleans, Dr. Preterre, formerly a partner of Dr. Fowler; also Dr. Stevens has been here for a long time, and his firm has done the largest business on this side of the ocean. He has a magnificent office; his collection of paintings cost him, I am told, one hundred thousand dollars; the amount of his busines is ($400,000) four hundred thousand dollars a year. Every branch of our profession in Paris is far behind us; the French are a receiving people, not a giving one, they do not appreciate fine work, quackery in the dental line by men calling themselves dentists; there is in this great city dentists at the present time who are ignorant of the use of gold foil, and say that it is not equal to amalgam, and these pretenders have a large practice, and every one has his mouth wash and in fact you will find it in all the shop windows on the *Boulevards* endorsed by the medical societies of this city, and an immense business is done in this line and a great deal of it is sent to our country. Amalgam, plain teeth, natural teeth, ivory and india rubber are chiefly used, and the profession is at a low standing; the American dentists have a good name, but I am sorry to say have done nothing to elevate the profession in the least, and there is some here who are able to do a great deal but they think nothing of the profession, except so far as it serves them to make money. It is, however, to be noticed, that American dentists are more numerous here than in any city of Europe. Paris is the great centre of everything (but dental societies,) the profession has not here the same advantage as to fees as in London. French people count francs instead of pounds, it has two dental depots, and one very good that any city might be proud of, it is well stocked with all that appertains to the dental line; the name of the depot is The Central Depot.

I am indebted to Dr. Preterre for taking me to the different hospitals and to the manufactory of teeth, (by the way being good plain teeth,) also for showing me his improved obturator, he deserves a great deal of credit for all the trouble and expense he has gone to by producing this useful and fine work, the construction of it is very simple, it has the advantage over all others of keeping itself perfectly clean; though simple, this obturator is complete, it is not clasped to any tooth but held by a spring which fastens over the gum; I have seen at the hospital a man who had the right upper maxillary taken away; Dr. Preterre inserted a piece of work restoring the contour of the face with his obturator which was a perfect success, and the patient after this fine operation was able to speak, whistle and sing perfectly well and with the case out of his mouth, he can not be understood; I felt like I wished to give a glorious shout for our profession; he spares neither time, work or money for his improvement, he has operated in hundreds of cases and a great many *free.* I have had the pleasure of seeing a great variety of interesting cases which have been successfully treated, his office contains hundreds of interesting cases which is open to all. I may say I have seen at least thirty different kinds of obturators since my sojourn in Paris; this improvement is endorsed by the first surgeons and medical men of the country. He is also preparing a work containing the description of all the cases he has had with excellent illustrations; his manner of regulating is also very interesting; he is preparing a case which he intends to present to the Ohio College.

As to any of my friends coming to this country for the purpose of practicing his profession, if he has business at home STAY THERE; *America suits me*; but, on the other hand, if he has plenty of cash and wants to cut his eye teeth, this is the country, the expense of living is much greater than in our own country, rent is higher and you must be up two or three pair of stairs and no water except what is brought up by hand, and now my friend I hope you will excuse me for intruding on your time; but, I can not remain here and not let you know what was going on on this side, if you choose to use this I hope you will cut and prune it to your liking, hoping to hear from you.

I am ever yours,

H. T. McKELLOPS.

